# Genetic Innovation in Vertebrates: Gypsy Integrase Genes and Other Genes Derived from Transposable Elements

**DOI:** 10.1155/2012/724519

**Published:** 2012-08-13

**Authors:** Domitille Chalopin, Delphine Galiana, Jean-Nicolas Volff

**Affiliations:** Institut de Génomique Fonctionnelle de Lyon, Université de Lyon, Ecole Normale Supérieure de Lyon, CNRS, Université Lyon 1, 69364 Lyon Cedex 07, France

## Abstract

Due to their ability to drive DNA rearrangements and to serve as a source of new coding and regulatory sequences, transposable elements (TEs) are considered as powerful evolutionary agents within genomes. In this paper, we review the mechanism of molecular domestication, which corresponds to the formation of new genes derived from TE sequences. Many genes derived from retroelements and DNA transposons have been identified in mammals and other vertebrates, some of them fulfilling essential functions for the development and survival of their host organisms. We will particularly focus on the evolution and expression of Gypsy integrase (*GIN*) genes, which have been formed from ancient event(s) of molecular domestication and have evolved differentially in some vertebrate sublineages. What we describe here is probably only the tip of the evolutionary iceberg, and future genome analyses will certainly uncover new TE-derived genes and biological functions driving genetic innovation in vertebrates and other organisms.

## 1. Introduction

For a long time, transposable elements (TEs) have been considered as pure selfish and junk elements parasiting the genome of living organism [1, 2]. These sequences are able to “move”, that is, to insert into new locations within genomes. This phenomenon is called transposition. Retroelements use retrotransposition, that is, the reverse transcription of an RNA intermediate and integration of the cDNA molecule produced, to generate new copies of themselves within genomes (copy-and-paste mechanism). This mechanism directly increases the copy number of the element. Among protein-coding autonomous retroelements, distinction is generally made between elements with long terminal repeats (LTRs: LTR retrotransposons and retroviruses) and retroelements without LTRs (non-LTR retrotransposons or LINE elements). Retroviruses and LTR retrotransposons are mainly distinguished by the presence versus absence of an envelope gene, which encodes a protein necessary for virus entry into the target cell. After germ line infection, reverse-transcribed retrovirus genomes can be integrated into the host genome and transmitted through vertical inheritance to the host progeny [3]. Such sequences, called endogenous retroviruses, are generally inactivated by mutations. Gain or loss of the envelope gene can transform a retrotransposon into a retrovirus, and *vice versa* [4, 5]. The second large category of TEs, DNA transposons, generally excises from their original insertion site and reintegrate into a new location (cut-and-paste mechanism). For most DNA transposons, transposition is catalyzed by an enzyme called transposase [6]. Finally, noncoding nonautonomous elements using for their transposition proteins encoded by autonomous sequences exist for both retroelements and DNA transposons.

Despite the deep-rooted vision of junk DNA, there is growing evidence that TEs are more than simple genome parasites. Particularly, they have been shown to serve as a genomic reservoir for new regulatory and coding sequences allowing genetic innovation and organismal evolution. A fascinating facet of the roles of TEs in evolution is their ability to be “molecularly domesticated” to form new cellular protein-coding genes [7, 8]. TE-encoded proteins have properties that can be of interest for host cellular pathways. They can bind, copy, cut, process, and recombine nucleic acids, as well as modify and interact with host proteins. There are many cases of TE-derived genes fulfilling important functions in plants, fungi, and animals, including vertebrates (for review, [8, 9]). We will present here several prominent examples of vertebrate genes formed from TE-coding sequences during evolution, with more emphasis on Gypsy integrase (*GIN*) genes that we have analyzed in different fish species.

## 2. Genes Derived from Retroelements

### 2.1. Gag-Derived Genes

Several multigenic families have been formed from different events of molecular domestication of the *gag* gene of Ty3/Gypsy elements, a super family of LTR retrotransposons active in fish and amphibians but extinct in mammals [9, 10]. The *gag* gene encodes a structural protein with three functional regions: the matrix (MA) domain playing a role in targeting cellular membranes, the capsid (CA) domain involved in interactions with other proteins during particle assembly, and the nucleocapsid (NC), which binds to viral RNA genomes through zinc fingers.

One *gag*-related gene family is called *Mart*. This gene family is mammal specific and constituted by 12 genes in human [11]. Most *Mart* genes are found on mammalian X chromosome, suggesting an initial event of molecular domestication on the X, followed by serial local duplication events that subsequently extended this gene family. All *Mart* genes have retained from the original *gag* sequence an intronless open reading frame. Some of them still encode the ancestral Gag zinc finger, suggesting nucleic acid binding properties for the protein. Two autosomal *Mart *genes, *PEG10* (*Mart2)* and *PEG11/Rtl1* (*Mart1*), are subject to genomic imprinting and are expressed from the paternal allele [12, 13]. This epigenetic regulation has been proposed to be derived from a defence mechanism repressing the activity of the ancestral retrotransposon before domestication [14]. At least two *Mart* genes, *PEG11/Rtl1* (*Mart1*) and *PEG10* (*Mart2)*, have essential but nonredundant functions in placenta development in the mouse [15, 16]. *PEG10 *and other *Mart* genes might also control cell proliferation and apoptosis, with possible involvement in cancer ([8] and references therein).

Another mammalian gene family derived from a LTR retrotransposon *gag* gene is called *Ma* or *Pnma* (paraneoplastic Ma antigens) [17]. Fifteen *Ma/Pnma* genes are present in the human genome, most of them being located on the X chromosome as observed for *Mart* genes. Some Ma proteins are expressed by patients with paraneoplastic neurological disorders and might be targeted by autoimmune response leading to progressive neurological damage [18]. Several Ma proteins are also involved in apoptosis, including Ma4 (Pnma4/Map1/Maop1) and Ma1/Pnma1 [19, 20].

A third family is the SCAN domain family. This family is constituted of DNA binding proteins with an N-terminus region called the SCAN domain, which is derived from the Gag protein of a Gmr1-like Gypsy/Ty3 retrotransposon [21–24]. The SCAN family is vertebrate specific, with approximately 70 and 40 members in human and mouse, respectively. Several SCAN proteins have been shown to be transcription factors regulating diverse biological processes such as hematopoiesis, stem cell properties, or cell proliferation and apoptosis (for review [21]).

Finally, other *gag*-related genes are present in mammalian genomes [10]. One of them, *Fv1*, is of retroviral origin and controls replication of the murine leukaemia virus in the mouse [25].

### 2.2. Envelope-Derived Genes

During mammalian evolution, retroviral envelope genes have been domesticated several times independently to generate genes involved in placenta development [26]. These genes, derived from endogenous retroviruses, encode proteins called syncytins. Syncytins mediate the fusion of trophoblast cells to form the syncytiotrophoblast layer, a continuous structure with microvillar surfaces forming the outermost foetal component of the placenta [27]. Two syncytin genes of independent origins encoding placenta-specific fusogenic proteins are present in human and other simians (Syncytin-1 and -2, [28]) as well as in rodents (Syncytin-A and Syncytin-B, [29]). Independent Syncytin genes are also found in rabbit [30], guinea pig [31], and Carnivora [32], indicating multiple convergent domestication of *env*-derived Syncytin genes in different mammalian sublineages. Some Syncytins might be involved in other biological processes. For example, human Syncytin-1 plays a role in osteoclast fusion, neuroinflammation, and possibly multiple sclerosis [33, 34]. 

Other retroviral *env*-derived open reading frames are present in vertebrate genomes; but intensive work is required to determine their functions. Some of them might confer resistance to viral infection, as shown for the *Fv-4* locus. This locus, containing an entire ecotropic murine leukemia virus (MuLV) *env* gene, controls susceptibility to infection by MuLV [35].

### 2.3. Other Retroelement-Derived Genes

In mammals, a gene called *CGIN1* is partially derived from the integrase gene of an endogenous retrovirus. The integrase gene has been fused 125–180 million years ago to a duplicate of the cellular gene *KIAA0323*. A role of *CGIN1* in resistance against retroviruses has been proposed [36]. 

Several genes with homology to retroelement aspartyl protease genes are present in vertebrate genomes. One of them, a gene encoding a protein called SASPase, is necessary for the texture and hydration of the stratum corneum, the outermost layer of the epidermis [37].

Finally, the telomerase, the reverse transcriptase extending the ends of linear chromosomes in vertebrates and other eukaryotes, might be derived from a retroelement [38].

## 3. Genes Derived from DNA Transposons

Many examples of genes derived from transposase genes from diverse subfamilies of DNA transposons have been described in vertebrates and other organisms [8, 39, 40]. One well-studied example is the recombination-activating protein Rag1, which together with Rag2 catalyzes the V(D)J somatic site-specific recombination responsible for the formation and diversity of genes encoding immunoglobulins and T-cell receptors in jawed vertebrates. *Rag1* has been formed from the transposase of a Transib DNA transposon, and the V(D)J recombination signal sequences recognized by Rag1 might be derived from the transposon ends bound by the ancestral transposase [41].

The mammal-specific gene *CENP-B* encodes a Pogo transposase-derived protein that controls centromere formation depending on the chromatin context [42]. Interestingly, an independent event of molecular domestication of Pogo transposase also led to the formation of centromeric proteins in fission yeast [43]. In yeast, CENP-B-like proteins restrict the activity of retrotransposons and promote replication progression at forks paused by retrotransposon LTRs [44, 45]. Other genes are derived from Pogo-like transposons in mammals [46]. One example is the *Jerky* gene, which encodes a brain-specific mRNA-binding protein that may regulate mRNA use in neurons [47].

Similarly, several examples of genes derived from hAT transposases have been found in mammals, some of them having been fused to zinc finger domains [46]. Some hAT transposase-related proteins work as transcription factors. One of them, ZEBD6/MGR, negatively regulates *IGF2* expression and muscle growth. Indeed, it has been shown that mutation in a regulatory sequence prohibiting ZEBD6/MGR binding leads to *IGF2 *upregulation and enhanced muscle growth in commercially bred pigs [48, 49].

In primates, the gene encoding the Metnase/SETMAR protein has been formed through fusion of the transposase gene of a Mariner transposon with a SET histone methyltransferase gene. Metnase/SETMAR is a DNA binding protein with endonuclease activity that promotes DNA double-strand break repair through nonhomologous end joining (NHEJ) [50, 51].

Several genes derived from PiggyBac-like transposons have been detected in human and other vertebrates [52]. One of them, *PGBD3*, serves as an alternative 3′ terminal exon for the Cockayne Syndrome B (CSB) gene, leading to the expression of a CSB-transposase fusion protein [53]. At least one Harbinger transposon-derived gene, *HARBI1*, encoding a predicted nuclease, is present in mammals, birds, amphibians, and fish [54]. Likewise, genes derived from a new type of DNA transposon called Zisupton have been identified in fish and other vertebrates [55]. Finally, mammalian and bird genomes possess at least one gene clearly derived from a P transposon; additional vertebrate genes like *THAP9* encoding proteins with a THAP domain might be also related to P-like transposases [8, 40, 56–61].

## 4. Gypsy Integrase Genes: Data from Fish

Two vertebrate genes with unknown functions, *GIN1* and *GIN2 (Gypsy Integrase 1 and 2), *encode proteins showing significant homologies to integrases encoded by LTR retrotransposons [62, 63]. Further analyses showed that both genes have been formed from GIN transposons, a new family of metazoan DNA transposons with a transposase that shows strong similarities with LTR retrotransposon integrases [64]. *GIN1,* which shows similarities with GINO transposons from *Hydra magnipapillata*, is present in mammals, birds, and reptiles, suggesting a molecular domestication event at the base of the Amniota ca. 300 million years ago. Mammalian GIN1 proteins have conserved amino-acid residues necessary for integrase activity. Using our own analyses, we will now particularly focus on the *GIN2* gene. We provide here updated *GIN2* structural and phylogenetic analyses using new vertebrate sequences and present first expression data for this gene in fish. 

**Figure 1:**
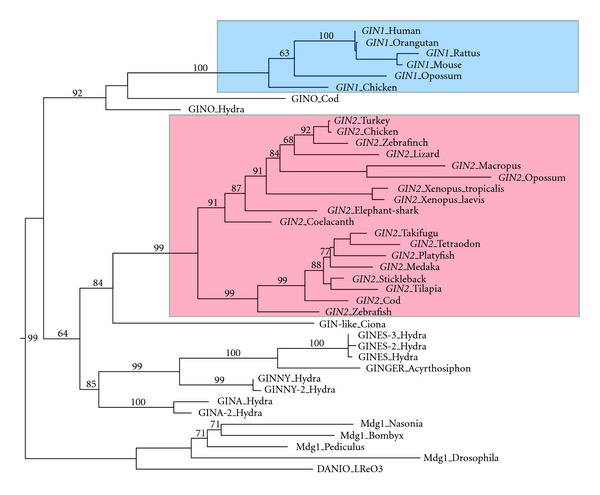
Molecular phylogeny of GIN proteins. Phylogenetic tree based on a 352 amino-acid integrase alignment. Protein sequences were aligned with clustalW and phylogenetic tree was constructed using maximum likelihood from PhyML package (optimized default bootstrap) [65]. Sequences were recovered from NCBI and Ensembl or predicted from genome sequences. Accession numbers and sequence alignments are available upon request.

**Figure 2:**
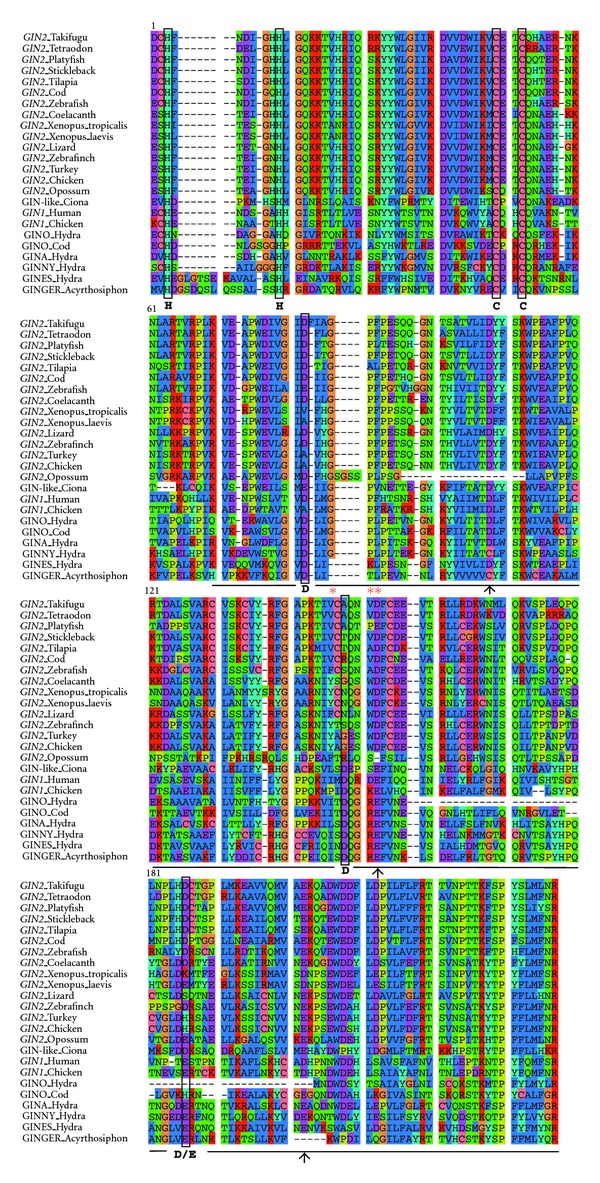
Sequence alignment of predicted GIN2-related proteins. HHCC zinc finger and integrase-like domain of *GIN2* were aligned using clustalW [66]. The black line indicates the position of the integrase-like domain. HHCC and DDE motifs are shown in black boxes and GPF motif is highlighted by red asterisks. Arrows indicate alternative conserved D/E residues in GIN2 sequences.

**Figure 3:**
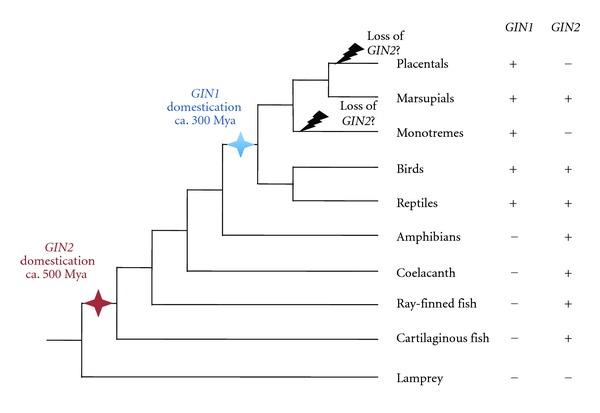
One possible scenario for the evolution of *GIN* genes in vertebrates. The two molecular domestication events are highlighted by blue and red stars, having led to the formation of *GIN1* and *GIN2, *respectively. Presence (+) or absence (−) of *GIN1* and *GIN2* in the different lineages is indicated.

*GIN2 *is present in several fish species, as well as in cartilaginous fish (elephant shark), coelacanth, amphibians, birds, reptiles, and marsupials, but neither in monotremes nor in placental mammals [63] (Figures 1, 2, and 3). Furthermore, *GIN2* was not detected in lamprey. Hence, the molecular domestication event having led to the formation of *GIN2* might have taken place before the divergence between tetrapods/bony fish and cartilaginous fish around 500 million years ago, with subsequent loss in monotremes and placental mammals. The formation of *GIN2* might even be older, since potentially domesticated GIN-like sequences related to *GIN2* have been detected in the urochordates *Ciona savignyi* and *C. intestinalis *[63]. Phylogenetic analysis suggests that *GIN2* is derived from GINA transposons, which are *bona fide* transposable elements in *Hydra magnipapillata *(Figure 1). This suggests that *GIN1* and *GIN2* have been formed through two independent molecular domestication events, one at the base of Amniota and the other in a more ancient vertebrate ancestor (Figure 3).

**Figure 4:**
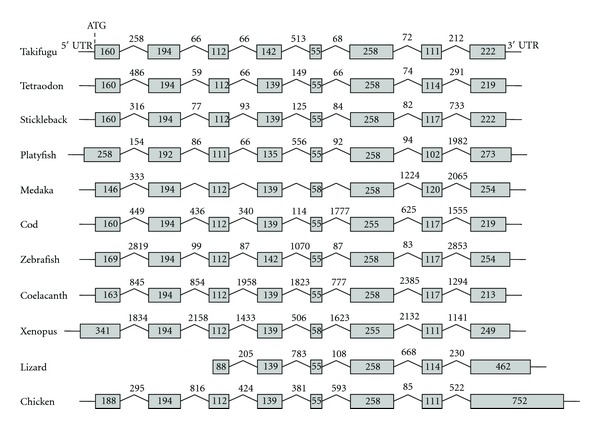
Exon-intron structure of *GIN2* genes in fish and other vertebrates. Exons are represented by grey boxes, introns by broken lines. Exon/intron sizes are given as base pairs.

**Figure 5:**
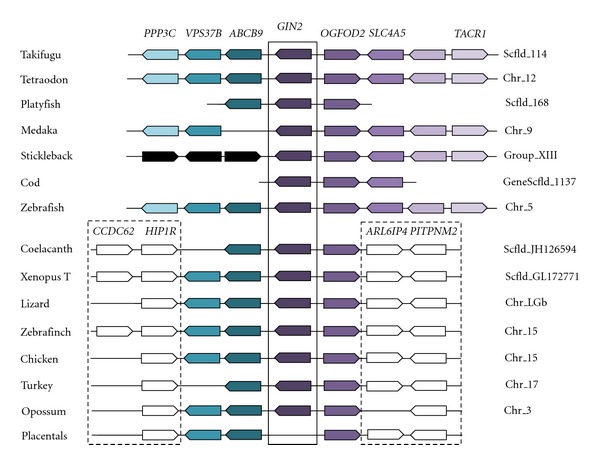
Comparison of *GIN2*-containing genomic regions in vertebrates. Synteny analysis was performed using Ensembl (http://www.ensembl.org/index.html), Genomicus (http://www.dyogen.ens.fr/genomicus-66.01/cgi-bin/search.pl), and BLAST analysis (http://blast.ncbi.nlm.nih.gov/Blast.cgi). Genes represented by white boxes are not found in this region in fish. Black boxes from stickleback represent different genes from ABCB9, VPS37B, and PPP3C in other fish. Their Ensembl accession numbers are from right to left: ENSGACG00000013652, ENSGACG00000013659, and ENSGACG13660.

After domestication, the HHCC zinc finger present in the ancestral integrase has been maintained, suggesting ability to bind to DNA or RNA (Figure 2). Conservation of the important catalytic triad (DDE, aspartic acid/aspartic acid/glutamic acid) of the integrase is less obvious. While this motif has been proposed to be conserved in GIN1, this is not the case for GIN2 based on a published alignment with sequences from GIN-related transposases [63] (Figure 2). As shown in Figure 2, the first aspartic acid residue is present in most species but absent from amphibians and birds. However, multiple sequence alignment revealed an aspartate conserved in all GIN2 and GIN1 sequences ca. 20 amino-acids downstream. The second aspartic acid residue is not found in GIN2 but an aspartate is conserved four amino acids away in all GIN2 sequences except for opossum. Finally, the glutamic acid residue is found only in several species and substituted by an aspartate in fish; but a conserved glutamate is detected 16 amino acids away. Hence, the question of the functionality of GIN2 as an integrase remains open and should be definitely answered through functional analyses. A third domain with unknown function called GPY/F [64, 67] is also detected in GIN proteins, but in some cases the phenylalanine residue is replaced by a leucin. *GIN2* contains eight protein-coding exons, with an exon-intron structure well conserved in fish and other vertebrates (Figure 4). Some introns might be derived from the ancestral transposon; others might be the result of events of intronization after molecular domestication. *GIN2* is located in the same orthologous genomic region between *OGFOD2* and *ABCB9* in marsupials, birds, reptiles, and fish, confirming that this gene does not correspond to a mobile sequence (Figure 5).

Expressed sequence tag (EST) analysis indicated that *GIN2* is expressed in different adult tissues and developmental stages in chicken: brain (accession number: CN219658), liver (BG713188), head (BU225420), embryonic tissue (BU210425), limb (BU256599), small intestine (BU297502), muscle (BU437928), and ovary (BU447634). Only ESTs from the whole body are available for *Xenopus*. Few ESTs are also found in zebrafish: muscle (CT684014), gills (EB908574), reproductive system (BI867074), and eye (BI879358).

**Figure 6:**
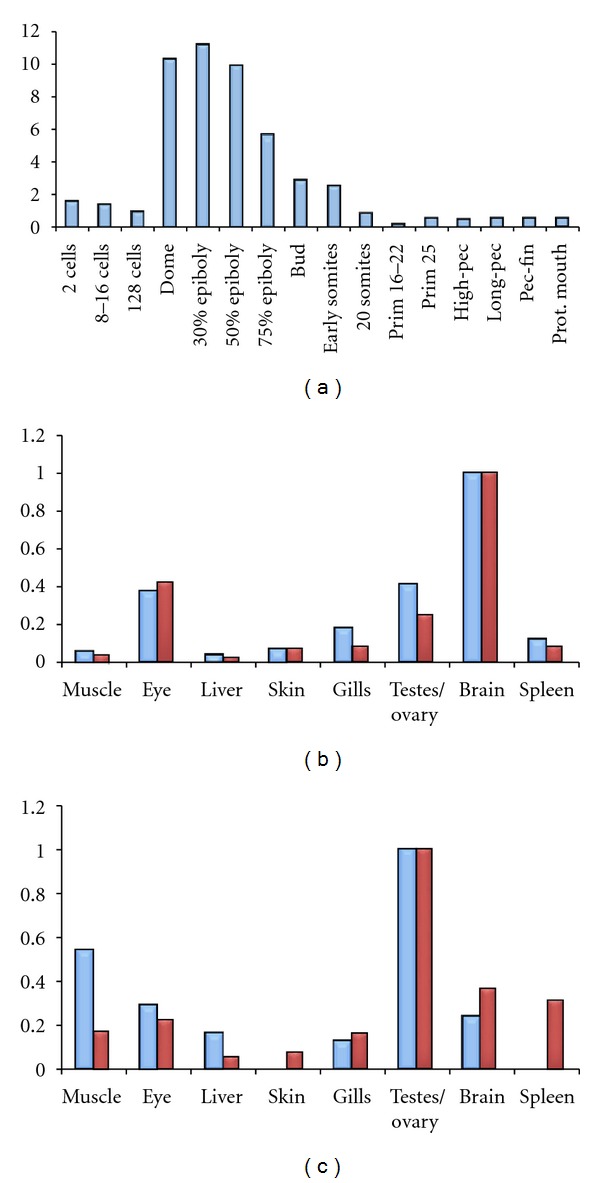
qPCR expression analysis of *GIN2* in zebrafish and platyfish. (a) Expression pattern of *GIN2* during embryonic development in zebrafish. (b) Expression pattern of *GIN2* in adult organs of zebrafish. (c) Expression pattern of *GIN2* in adult organs of platyfish. Multiple RNA extractions using different individuals were performed leading to independent sets of cDNA. Two independent sets and three independent sets of cDNA were tested for embryonic stages and adult organs, respectively. For all sets and for each sample of cDNA, qPCR reaction was done three times (triplicate). One representative experiment is shown with blue bars for male samples and red bars for female samples. *GIN2* expression was normalized using three housekeeping genes: *RPL7*, *beta-actin* and *EF1-alpha*. Analyses were done using the ΔΔCt method [55]. mRNA extractions were done using Trizol and reverse transcription steps were carried out using Fermentas kit. Finally, qPCR was performed using a Bio-Rad kit at the following step: 40 cycles of 94°C and 55°C. Primer sequences are available upon request.

To determine more precisely *GIN2* expression pattern in fish, quantitative real-time PCR was performed on different embryonic developmental stages in zebrafish (*Danio rerio*), as well as on adult tissues from zebrafish and platyfish (*Xiphophorus maculatus*) (Figure 6). During zebrafish embryogenesis, *GIN2* expression level strongly increases from the dome stage and progressively decreases until the end of somite stages. This result suggests that *GIN2* possibly plays a role during gastrulation. Gastrulation, which is characterized by morphologic movements of involution and extension, starts at the beginning of the epiboly to finish at bud stage [68]. In adult zebrafish, the higher level of expression for *GIN2* was observed in brain, followed by gonads and eyes. In contrast, *GIN2* expression was maximal in gonads in the platyfish (Figure 6).

To conclude, our analysis integrates data from several newly sequenced vertebrate genomes, particularly teleostean and cartilaginous fishes as well as coelacanth, in order to better understand the distribution and evolutionary history of *GIN* genes. Since *GIN2* is apparently not present in lamprey, we propose that *GIN2* was formed before the divergence between cartilaginous and ray-finned fish about 500 million years ago (Figure 3). We also provide the first expression data for *GIN2* in fish particularly supporting a function in gastrulation during zebrafish embryogenesis.

## 5. Conclusion

At first glance, transposable elements were considered as “junk” DNA with no important functions for genomes and organisms. Today, nobody can deny the importance of transposable elements during evolution in terms of innovation power, particularly through molecular domestication events. Domesticated elements are *bona fide* cellular genes derived from transposable element sequences encoding for example integrases, transposases, Gag proteins, or envelopes. After domestication, TE-derived genes have lost their ability to transpose through the elimination of sequences such as long terminal repeats, terminal-inverted repeats, or other open reading frames and protein domains essential for transposition. Elimination of such sequences might occur by genetic drift or might even be selected for transposition or retrotransposition of a domesticated sequence might change its copy number and pattern of expression. Many domesticated sequences have important functions, for example in cell proliferation. Transposition of such a gene might have strongly deleterious consequences for the host, for instance cancer. It might, therefore, be important to immobilize TE-derived genes at fixed position within a genome to control their expression.

In vertebrates, many TE-derived genes are mammal specific, suggesting that molecular domestication probably played an important role in the evolution of this specific sublineage. Accordingly, many domesticated sequences are involved in placenta formation. Other TE-derived genes like *GIN2* are present in some vertebrate sublineages but absent from mammals. In birds, reptiles, amphibians, and fish, domesticated sequences might be more difficult to identify due to the concomitant presence of active TEs within genomes. Availability of additional genome sequences will probably allow the identification of many TE-derived genes specific of these sublineages that contribute to diversification within vertebrates.

We focused on *GIN* genes, a pair of ancient vertebrate domesticated genes for which no function has been identified so far. Both *GIN1* and *GIN2* are derived from GIN transposons that themselves gained their transposase from the integrase of LTR retrotransposons.

*GIN1* was detected in mammals, birds, and reptiles, indicating that it was formed in a common ancestor of Amniota ca. 300 million years ago [63]. *GIN2* might be even older, since it was detected in tetrapods, bony fish, and sharks, and possibly in urochordates. The presence of both genes over such long periods of evolution is suggestive of important, so far unknown conserved functions in vertebrates. *GIN2 *was lost in a common ancestor of monotremes and placental mammals, suggesting that either GIN2 function was not essential anymore, or that this function is fulfilled now by GIN1 in these sublineages.

The evolutionary scenario having led to the formation of *GIN1* and *GIN2* remains unclear. Presence of conserved intron positions [63] suggests a unique origin followed by duplication and intron gain in a common ancestor of *GIN1* and *GIN2 *(paralogy). In this case, *GIN1* would have been lost among others in fish. Alternatively, *GIN1* and *GIN2 *might have been generated from two independent events of molecular domestication, as suggested by the close phylogenetic relationship of *bona fide* GIN transposons with each of both genes (Figure 1). Presence of introns at conserved positions might in this case reflect intron conservation between ancestral GIN transposons at the origin of both molecular domestication events.

GIN1 and GIN2 functions might be related to the binding to DNA or RNA, since both proteins have conserved the HHCC zinc finger present in the ancestral integrase. Conservation of the integrase activity appears possible but must be tested through functional assays. In fish, *GIN2* is particularly expressed in brain and gonads; its expression pattern during zebrafish embryogenesis suggests a role during gastrulation. Functional analysis in fish will provide important insights into the biological function of *GIN2* in vertebrates.

Taken together, data on *GIN* and other TE-derived genes support the important role of molecular domestication as a driver of genetic innovation during evolution. What we have presented here probably only represents the tip of the evolutionary iceberg. There is no doubt that future genome comparisons and functional gene analyses will uncover new domesticated genes and novel biological functions essential for the diversification of vertebrates and other living organisms.
